# Social determinants of COVID-19 incidence and outcomes: A rapid review

**DOI:** 10.1371/journal.pone.0248336

**Published:** 2021-03-31

**Authors:** Tara L. Upshaw, Chloe Brown, Robert Smith, Melissa Perri, Carolyn Ziegler, Andrew D. Pinto

**Affiliations:** 1 Upstream Lab, MAP Centre for Urban Health Solutions, Li Ka Shing Knowledge Institute, St. Michael’s Hospital, Toronto, Canada; 2 Translational Research Program, Faculty of Medicine, University of Toronto, Toronto, Canada; 3 Undergraduate Medical Education, Faculty of Medicine, University of Toronto, Toronto, Canada; 4 Institute of Health Policy, Management and Evaluation, Dalla Lana School of Public Health, Toronto, Canada; 5 Department of Family and Community Medicine, Faculty of Medicine, University of Toronto, Toronto, Canada; 6 Dalla Lana School of Public Health, University of Toronto, Toronto, Canada; 7 Health Sciences Library, Unity Health Toronto, Toronto, Canada; 8 Department of Family and Community Medicine, St. Michael’s Hospital, Toronto, Canada; SUNY Downstate: SUNY Downstate Health Sciences University, UNITED STATES

## Abstract

Early reports indicate that the social determinants of health are implicated in COVID-19 incidence and outcomes. To inform the ongoing response to the pandemic, we conducted a rapid review of peer-reviewed studies to examine the social determinants of COVID-19. We searched Ovid MEDLINE, Embase, PsycINFO, CINAHL and Cochrane Central Register of Controlled Trials from December 1, 2019 to April 27, 2020. We also searched the bibliographies of included studies, COVID-19 evidence repositories and living evidence maps, and consulted with expert colleagues internationally. We included studies identified through these supplementary sources up to June 25, 2020. We included English-language peer-reviewed quantitative studies that used primary data to describe the social determinants of COVID-19 incidence, clinical presentation, health service use and outcomes in adults with a confirmed or presumptive diagnosis of COVID-19. Two reviewers extracted data and conducted quality assessment, confirmed by a third reviewer. Forty-two studies met inclusion criteria. The strongest evidence was from three large observational studies that found associations between race or ethnicity and socioeconomic deprivation and increased likelihood of COVID-19 incidence and subsequent hospitalization. Limited evidence was available on other key determinants, including occupation, educational attainment, housing status and food security. Assessing associations between sociodemographic factors and COVID-19 was limited by small samples, descriptive study designs, and the timeframe of our search. Systematic reviews of literature published subsequently are required to fully understand the magnitude of any effects and predictive utility of sociodemographic factors related to COVID-19 incidence and outcomes. PROSPERO: CRD4202017813.

## Introduction

In the year since SARS-CoV-2 was identified in Wuhan, China the coronavirus disease 2019 (COVID-19) pandemic has resulted in more than 96 million cases and over 2 million related deaths [1]. Although COVID-19 was initially deemed a “great equalizer” given universal susceptibility to this novel virus [2], reports emerged in late March 2020 that COVID-19 morbidity and mortality disproportionately impacted groups made vulnerable by policies that create and reinforce health disparities [3–6].

Preliminary analyses from the United States, Canada and the United Kingdom reported high rates of COVID-19 infections, hospitalizations and mortality in geographic regions with high densities of low-income and crowded households, and in locations where a high proportion of individuals were racialized [7–10]. Early epidemiological studies from the United States found that African-Americans had the highest mortality rates of any group, with Native Americans close behind [11–16]. The United Kingdom’s Office of National Statistics reported that Black males and females were respectively 4.2 and 4.3 times more likely to die from COVID-19, when compared to White individuals adjusting for age [17]. People in congregate settings, including prisons and homeless shelters, and long-term institutional care facilities also appeared to be at higher risk for infection and worse outcomes once infected [18–20].

Infection rates and outcomes for infectious diseases are influenced by social factors [21]. For example, during the 2009 H1N1 pandemic, incidence was highest among those without access to paid sick leave, and racialized individuals and those experiencing high material deprivation were more likely to be admitted to intensive care units [22–24]. While there is a growing recognition that social factors have similarly influenced COVID-19, the synthesis of relevant research is limited. Given that an empirical understanding of the broader social determinants of COVID-19 could inform ongoing pandemic response efforts, we conducted a rapid review of early reports on the social determinants of COVID-19 infection, health service use and health outcomes.

## Methods

### Data sources and searches

We designed this rapid review using interim guidance from the Cochrane Rapid Review Methods Group [25] and registered the review protocol with PROSPERO (CRD42020178131). We searched Ovid MEDLINE, Embase, PsycINFO, CINAHL and Cochrane Central Register of Controlled Trials bibliographic databases (S1 File) from December 1, 2019 to April 1, 2020, updating once on April 16, 2020, and again on April 27, 2020. We used a combination of search terms for SARS-CoV-2 and COVID-19. Database searches were supplemented with manual searches of bibliographies of included studies, COVID-19 evidence repositories and living evidence maps [26–28], and report referrals solicited from research colleagues (Australia, Belgium, Canada, China, United States and United Kingdom) with expertise in health equity and population health (S2 File). Experts were contacted by email and asked to forward reports that fit within our inclusion criteria. They were welcomed to forward our request to others within their network. We included studies identified through supplementary sources up to June 25, 2020.

### Study selection

We included English-language peer-reviewed quantitative studies that used primary data to describe the social determinants of COVID-19 infection, health service use or health outcomes in adults (18 years and older) with a confirmed or presumptive diagnosis of COVID-19. We included analyses of surveillance data published by public health agencies, and excluded modelling studies, secondary analyses, news items, opinions and editorials. Using emerging reports on how social factors impacted COVID-19 and the framework of the World Health Organization Commission on the Social Determinants of Health [29], we focused on studies that reported participant race or ethnicity, income, education, employment, housing status, food security, and social isolation (S1 Table). We excluded studies that only reported age and biological sex of participants, as these relationships are well-described elsewhere [30].

### Data extraction and quality assessment

Data extraction was completed using a piloted form (S3 File) by two members of the study team (TLU and CB) and confirmed by another study team member (RS). We conducted quality assessment using the Mixed Methods Appraisal Tool (MMAT) [31]. The MMAT is a validated tool for appraising quality of quantitative randomized, quantitative non-randomized, quantitative descriptive, qualitative and mixed-methods studies included in mixed literature reviews [31, 32].

### Data synthesis and analysis

Given the heterogeneity of study designs, we did not conduct a pooled analysis and instead conducted a narrative synthesis [33, 34]. We organized article findings by related social determinants of health, and then by study design within each determinant category. If studies addressed more than one determinant, we described them in multiple categories.

## Results

**Fig 1:**
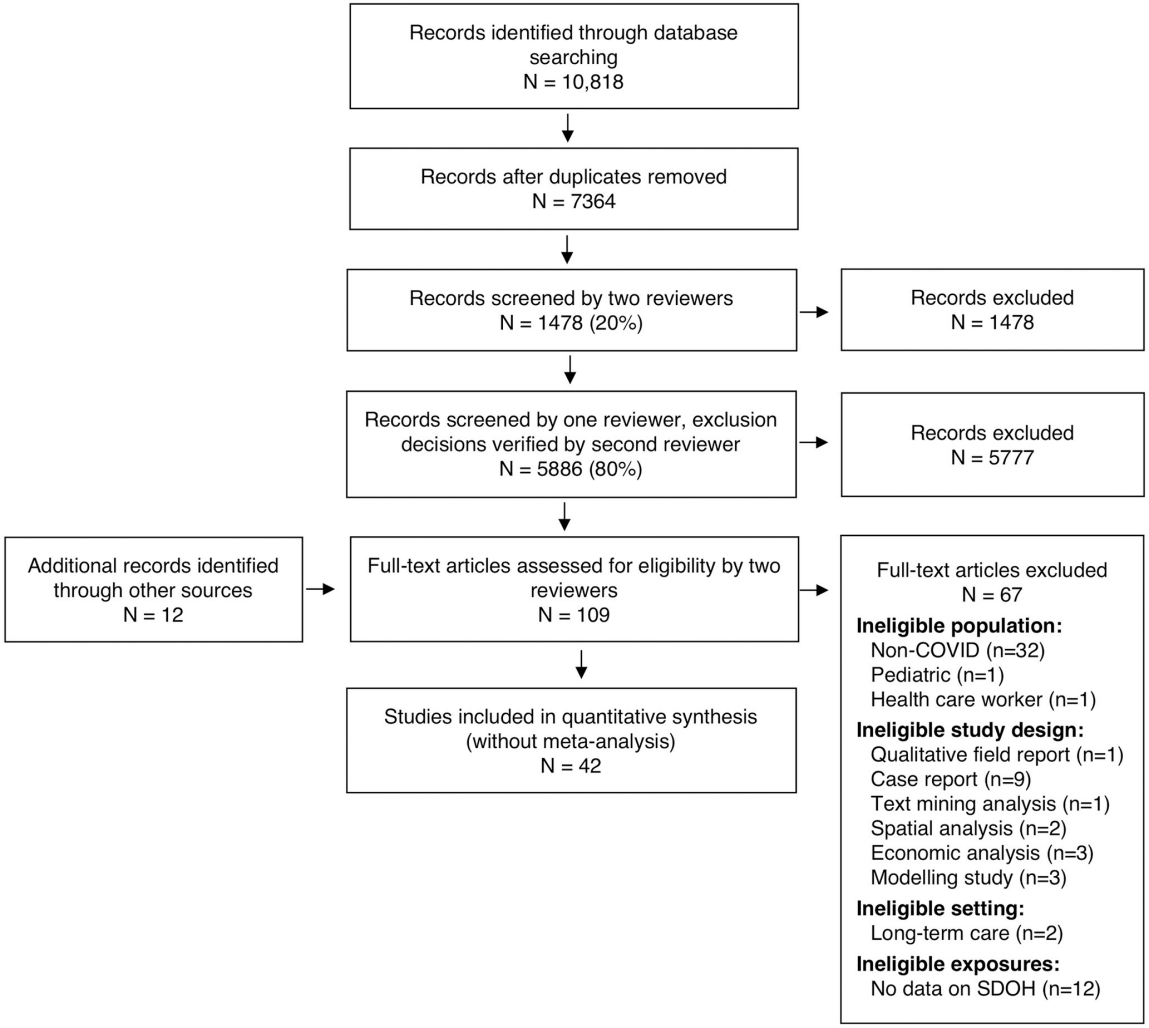
PRISMA flow diagram.

Of 7,376 records screened, (Fig 1), 42 articles met our inclusion criteria (Table 1), 12 of which were identified through supplementary sources. These studies were conducted in China (13) and high-income countries, including Australia (2), Singapore (2), Spain (1), the United Kingdom (2), the USA (21), and a group of European Union member countries. They included cross-sectional (*n* = 19), cohort (*n = 11*), case series (*n = 8*) and case-control (*n = 4*) designs. Of included studies, 23 reported participant race or ethnicity data, 16 on occupation, 5 on income, 2 each on education and social isolation, 1 on food security and 6 on housing status.

**Table 1:** Characteristics and summarized results of studies.

Author, (Year)	Country	Study Design; Sample Size (*N*)	Social Factors Examined	Summary of findings
**Cohort Design**				
Azar et al., (2020) [37]	California, United States	Retrospective cohort *N* = 14,036 (1052 cases)	Race/ethnicity, income, housing status	Examined disparities in COVID-19 related outcomes. Non-Hispanic Black were more likely to be admitted to hospital than non-Hispanic White (*OR* = 2.67, *95% CI*: 1.30–5.47, *p* < 0.01). COVID-19 patients with Medicaid /uninsured were more likely to be admitted to hospital than those with commercial insurance (Medicaid: *OR* = 2.13; *95% CI*: 1.24–3.68, *p*<0.01; Self-Pay/Unknown: *OR* = 2.19; *95% CI*: 1.03–4.36, *p*<0.05). COVID-19 patients residing in higher income neighbourhoods were less likely than those residing in lower income neighbourhoods to be admitted to hospital (High income, fourth quartile: *OR* = 0.55, *95% CI*: 0.33–0.91, *p*<0.05; High income, Third quartile: *OR* = 0.24, *95% CI*: 0.12–0.46, *p*<0.001). No statistically significant association between homelessness and hospital admissions.
Dai et al., (2020) [62]	Jiangsu, China	Retrospective cohort; *N* = 234	Occupation	Analyzed the chest CT and clinical characteristics of COVID-19 positive patients. 11.1% had no occupation, 11.1% were farmers, 4.7% were students, 1.3% were medical staff, and 24.5% were listed as other.
Fan et al., (2020) [58]	Gansu, China	Retrospective cohort; *N* = 54	Occupation	Examined characteristics of COVID-19 in two consecutive time waves. Majority of cases in first wave were laborers (29.0%). Majority of cases in second wave order were retirees (47.0%) (*p* = 0.009).
Garg et al., (2020) [11]	United States	Retrospective cohort *N* = 1,482	Race/ethnicity	Examined hospitalization characteristics of COVID-19 positive patients. Among the 39.1% of patients with available race and ethnicity data, the largest proportion were non-Hispanic White (45%), then 33.1% non-Hispanic black, 9.1% Hispanic, 5.5% Asian, 0.3% American Indian/Alaskan Native, and 7.9% other/unknown race.
Hastie et al., (2020) [35]	United Kingdom	Retrospective cohort *N* = 449	Race/ ethnicity, socioeconomic deprivation	Assessed whether Vitamin D concentration was associated with incidence of COVID-19. Compared to White individuals, Black and South Asian individuals were more likely to test positive for COVID-19 (Black: *OR* = 4.30, *95% CI*: 2.92–6.31, *p*<0.001; South Asian: *OR* = 2.42, *95% CI* = 1.50–3.93, *p*<0.001). Higher socioeconomic deprivation predicted COVID-19 positive status (highest vs. lowest Townsend quintile *OR* = 1.89; *95% CI* = 1.37–2.60; p-value<0.001).
Lechien et al., (2020) [47]	Europe (Belgium, France, Spain, Italy)	Prospective cohort *N* = 417	Race/ethnicity	Analyzed olfactory and gustatory dysfunction as a clinical presentation of mild to moderate COVID-19. 93.3% of patients were European, 0.2% Asian, 1.4% Black African, 2.2% Sub-Saharan African, 0.2% North American; and 2.6% South American.
Mehta et al., (2020) [56]	Ohio and Florida, United States	Retrospective cohort *N* = 18,472	Race/ ethnicity	Examined associations between ACE-inhibitors, ARB use and COVID-19 diagnosis. The majority of patients identified as White (69%).
Shi et al., (2020) [73]	Zhejiang, China	Retrospective cohort; *N* = 487	Occupation	Examined predictors of COVID-19 severity. Majority of cases were self-employed (45.0%) or worked in agriculture (28.7%). Bivariate analyses found statistically significant differences in low versus high severity groups by occupation (*p*<0.01).
Toussie et al., (2020) [41]	New York City, United States	Retrospective cohort *N* = 338	Race/ethnicity	Examined the association between clinical and chest radiography and COVID-19 related outcomes. Among positive cases (n = 338), 21% were White, 9% Asian, 34% Hispanic, 23% Black, and 13% unknown. The study found no statistically significant difference in primary outcomes (hospitalization, intubation, sepsis, prolonged length of stay, mortality) across race/ethnicity.
X. Wang et al., (2020) [63]	Wuhan, China	Retrospective cohort *N* = 80	Occupation	Analyzed clinical characteristics of medical workers who were COVID-19 positive. 51.3% of cases were among nurses, 29.8% were among doctors and 20.0% were among other medical workers.
Yan et al., (2020) [40]	San Diego, United States	Retrospective cohort *N* = 128	Race/ethnicity	Examined self-reported olfactory loss and clinical course for COVID-19 positive patients. COVID-positive admitted patients were 30.8% White, 11.5% Black, 26.9% Hispanic, 15.4% Asian, and 15.4% other/mixed. Race was not associated with anosmia or hospital admission.
**Case-Control Design**				
Nobel et al., (2020) [39]	New York City, United States	Case-control *N* = 516 (278 cases; 238 controls)	Race/ ethnicity	Assessed gastrointestinal symptoms of COVID-19 patients. OF the COVID-19 positive patients, 30% were White, 28% Black, 1.4% Asian, 41% other/unknown; 39% were Hispanic, 41% non-Hispanic, 21% other/unknown. No significant differences in COVID-19 positivity by race (p = 0.29) and ethnicity (p = 0.14).
Sun, Y et al., (2020) [38]	Singapore	Case- control *N* = 788 (54 cases; 734 controls)	Race/ethnicity	Assessed the relationship between ethnicity and COVID-19. Among those who were COVID-19 positive, 88.9% were Chinese (versus 75.3% in controls), 1.9% were Malay (7.9% controls), 9.3% were Indian (8.7% of controls), and 0% of cases were “other” ethnicity. No statistically significant differences found in COVID-19 status by ethnicity (*p* = 0.045).
Tolia, Chan and Castillo, (2020) [49]	United States	Case-control; *N* = 283 (29 cases; 254 controls)	Race/ethnicity	Assessed the characteristics of COVID-19 positive cases. Among those patients that tested positive, 69% were non-Hispanic White, 13.8% were Hispanic, 0% non-Hispanic Black, 6.9% non-Hispanic Asian/PI, and 10.2% other/mixed/unknown. Among those that tested negative, 18.5% were Hispanic, 55.5% non-Hispanic White, 5.1% Non-Hispanic Black, 9.8% non-Hispanic Asian/PI, and 11% other/mixed/unknown.
Yu et al., (2020) [66]	Wenzhou, China	Case-control *N* = 92 (11 cases; 62 controls)	Occupation	Assessed the occupational characteristics of COVID-19 patients. Majority of patients worked in the agriculture sector (48.9%), then self-employed workers (22.8%), employees (8.7%), 1retired (8.5%), and student (1.1%). No statistically significant relationship found between type of occupation and severity of the illness.
**Cross-Sectional Design**				
Baggett et al., (2020) [77]	United States	Cross-sectional *N* = 408	Race/ethnicity, housing status	Analyzed the incidence of COVID-19 within a homeless shelter. 36% of residents tested positive, the majority of which were White (47.2%), then Black/African-American (31.9%), Asian (2.8%), American Indian/ Alaskan Native (1.4%), Other (41.6%), and Multiple (2.1%) races; Hispanic/Latino (16.1%).
Burrer et al. (2020) [51]	United States	Cross-sectional *N* = 8,945	Race/ethnicity	Analyzed the characteristics of health care personnel (HCP) with COVID-19. Among 3801 HCP with available data on race, 72.0% were White, 21% Black, 5% were Asian, and 2% were other/multiple races. Among 3624 HCP with ethnicity data available, it was found that 90.0% were non-Hispanic/ Latino and 10% were Hispanic/Latino.
COVID- National Incident Room Surveillance Team, (2020) [44]	Australia	Cross-sectional *N* = 2,355	Race/ethnicity	Analyzed the prevalence of COVID-19 among Indigenous populations in Australia. 0.6% of cases were Aboriginal and Torres Strait Islander persons.
COVID-19 National Incident Room Surveillance Team, (2020) [45]	Australia	Cross-sectional *N* = 6,394	Race/ethnicity	Assessed characteristics of individuals who tested positive for COVID-19. 0.7% of cases were Aboriginal and Torres Strait Islander persons.
De Lusignan et al., (2020) [36]	United Kingdom	Cross sectional *N* = 3,802 (587 cases)	Race/ethnicity, socioeconomic deprivation, social isolation	Assessed risk factors for COVID-19. The likelihood of testing COVID-19 positive among Black were higher compared to White adults after adjustment (*OR* = 4.75, *95% CI* = 2.65–8.51). The odds of a positive test were lower in households with two to eight people, compared to single-person households in a univariate analysis (*p*<0.0001), but not in a multivariate analysis. People living in more socioeconomically deprived areas were more likely to test positive for COVID-19 (*OR* = 2.03, *95% CI*: 1.51–2.71, *p<*0.0001).
Dyal et al., (2020) [71]	United States	Cross-sectional *N* = 130,578 (4,913 cases)	Occupation	Assessed the incidence of COVID-19 among individuals working at 115 meat and processing facilities. Approximately 3.0% of individuals tested positive for COVID-19, 0.4% died.
Gold et al., (2020) [42]	Georgia, United States	Cross-sectional *N* = 305	Race/ethnicity, income	Examined the characteristics and clinical outcomes of COVID-19 positive patients. Among individuals with available data on race (97.4%), 83.2% were Black, 10.8% were non-Hispanic white, 2.7% were non-Hispanic Asian or Pacific Islander, and 3.4% were Hispanic. Majority of patients had private insurance (40.1%) or Medicare (33.4%); 10.9% had Medicaid, and 14.9% were uninsured.
Hasan & Narasimhan, (2020) [43]	United States	Cross-sectional *N* = 227	Race/ethnicity	Assessed the characteristics of COVID-19 patients. 46.3% were White, 22.5% were Black, 9.3% were Asian and 22.0% were either multi-racial or unknown.
Jia et al., (2020) [60]	Qingdao, China	Cross-sectional *N* = 11 clusters, 44 confirmed cases	Occupation	Analyzed characteristics of COVID-19 positive cases. Largest proportion of cases were employees (45.5%), followed by retirees (18.2%), unemployed (15.9%), medical staff (11.4%), and students (9.1%).
Laurencin & McClinton, (2020) [48]	Connecticut, United States	Cross-sectional *N* = 1726 cases	Race/ethnicity	Assessed characteristics of individuals who tested positive for COVID-19 in Connecticut. Of those with COVID-19, 60.8% were White (compared to 66.5% of population), 17.2% Black (12% of population), 2.9% Asian (4.0% of population), 15.9% Hispanic/Latinx (16.5% of population), 0.2% American Indian/ Alaska Native (0.6% of population), and 2.9% other. Among those who died, 76.7% were White, 14.4% Black, 6.7% Hispanic/ Latinx, and 2.2% Asian.
Li et al., (2020) [76]	China	Cross-sectional *N* = 182	Food security	Assessed the prevalence of malnutrition in elderly patients who had COVID-19. 52.7% were malnourished, 27.5% were at risk of malnutrition (50), and 19.8% were non-malnourished (*p* = 0.018).
Mosites et al., (2020) [69]	Boston, Seattle, San Francisco and Atlanta, United States	Cross-sectional *N*_*staff*_ = *313 ((*33 cases*) N*_*residents*_ = *1*,*292* (292 cases)	Housing status, occupation	Assessed COVID-19 incidence in homeless shelters. 25% of residents and 11% of staff were found to be positive.
Ouyang et al., (2020) [64]	Bejing, China	Cross-sectional *N* = 11 (patients)	Occupation	Measured immune response during COVID-19 disease progression. No statistically significant difference between the severity of the disease and occupation status or type. Majority of individuals (54.6%) were retired or farmers, with 83.3% of these groups experiencing severe COVID-19 symptoms.
Tobolowsky et al., (2020) [70]	Washington, United States	Cross-sectional *N*_*staf f*_ = *38 (*8 cases*) N*_*residents*_ = *195* (35 cases)	Housing status, occupation	Assessed the incidence of COVID-19 among residents and staff three homeless shelters: 18% of residents and 21% of staff were found to be positive.
Wang, R. et al., (2020) [61]	Fuyang, China	Cross-sectional *N* = 125	Occupation	Analyzed characteristics of COVID-19 positive cases. Majority of cases were employees (47.2%), followed by agricultural workers (20.8%). The lowest proportion of cases were students (6.4%) and retired individuals (2.4%).
Wallace, Hagan et al., (2020) [67]	United States	Cross-sectional *N* = 7,671	Housing status, occupation	Assessed the incidence of COVID-19 among residents and staff of correctional facilities. 4,893 COVID-19 cases were found among residents and 2,778 cases were found among staff members. 10% of residents were hospitalized and 2% died.
Wallace, Marlow et al., (2020) [68]	United States	Cross-sectional *N* = 742	Housing status, occupation	Aimed at assessing COVID-19 incidence among residents and staff of correctional facilities. Among residents, there were a total of 489 positive cases, 7.6% which were hospitalized and 2% which died. Among staff, there were a reported 253 cases, 7.5% which were hospitalized and 1.6% which died.
Xiao et al., (2020) [74]	China	Cross-sectional *N* = 170	Social capital, income, education	Assessed the impact of social capital on sleep quality and mental health of those in isolation due to COVID-19. 70.6% of subjects had a mid-monthly income between 5000–8000 yuan. 72.3% of patients had a college education. Higher social capital scores were significantly associated with lower anxiety and stress (structural equation model coefficients: anxiety, β = 0.619, *p*<0.001; stress, β = 0.327, *p*<0.001).
Zhang et al., (2020) [75]	China	Cross-sectional *N* = 205	Education	Assessed mental health outcomes of people impacted by COVID-19. Of those who had COVID-19 (n = 57), 30.9% had a junior-middle school education or less, 27.3% had senior middle school education, and 41.8% had a college education or more. There was no statistical difference in education level between those who experienced COVID-19 and members of the general public.
**Case Report/Series Design**				
Bangalore et al., (2020) [55]	United States	Case series *N* = 18	Race/ ethnicity	Examined ST-Segment Elevation in COVID-19 positive patients. Among those with ST-segment elevation myocardial infarction or noncoronary myocardial injury, 22% were White, 11% were Black, 50% were Hispanic, and 17% were Asian.
Blanco et al., (2020) [72]	Spain	Case series *N* = 5	Occupation	Assessed the COVID-19 incidence among individuals who were HIV positive. 40% were sex workers, among whom one was admitted to the ICU. 80% of participants identified as men who have sex with men.
Chu et al., (2020) [57]	China	Case series; *N* = 54	Occupation	Examine the risk of COVID-19 exposure and infection status among medical staff. Highest number of cases (72.2%) were found among non-emergency clinical departments, which also had the highest disease severity rates (69.8%).
Goyal et al., (2020) [52]	New York City, United States	Case series *N* = 393	Race/ethnicity	Analyzed clinical characteristics of COVID-19 patients. They found the majority of cases were in non-White individuals (37.4% of patients were reported as White). Of those who required invasive mechanical ventilation, 35.4% were White.
Pung et al., (2020) [46]	Singapore	Case series *N* = 36	Race/ethnicity	Analyzed characteristics of three clusters of COVID-19. 94% of cases were Chinese and 76% were Singaporean.
Richardson et al., (2020) [53]	New York City, Long Island and Westchester County, United States	Case series *N* = 5,700	Race/ethnicity	Assessed the characteristics of COVID-19 positive patients. For patients with available race data (n = 5441), 39.8% were White, 22.6% were African-American, 8.7% were Asian, and 28.9% were other/multiracial. For patients with available ethnicity data (n = 5341), 77% were non-Hispanic and 30% were Hispanic.
Sun, H et al., (2020) [50]	New York City, United States	Case series *N* = 30	Race/ethnicity	Analyzed the characteristics of COVID-19 positive cases. Majority of cases identified as Hispanic (66.7%) followed by White (13.3%) and then Black (6.7%).
Wang, L. et al., (2020) [65]	Liaocheng, China	Case series *N* = 26	Occupation	Assessed characteristics of COVID-19 patients. The majority of individuals were retail workers (61.5%), followed by retirees (15.4%), students (11.5%), agricultural workers (7.7%), and self-employed (3.9%). 11 of the 16 retail staff patients were working at the same supermarket.

### Race or ethnicity

Twenty-three studies (55%) reported participant race or ethnicity data. Three large studies found statistically significant differences in COVID-19 infection incidence and hospitalization outcomes by race or ethnicity [35–37]. A prospective analysis of UK Biobank data (*n* = 348,598; 499 cases) found that compared to White individuals, Black and South Asian individuals were more likely to test positive for COVID-19 after adjustment for socioeconomic, lifestyle and health-related factors (Black: *OR* = 4.30, *95% CI*: 2.92–6.31, *p*<0.001; South Asian: *OR* = 2.42, *95% CI* = 1.50–3.93, *p*<0.001) [35]. Another UK study (*n* = 3,802; 587 cases) observed similar increases in the likelihood of testing COVID-19 positive among Black compared to White adults after adjustment for potential confounders (*OR* = 4.75, *95% CI* = 2.65–8.51) [36]. In California, USA, a retrospective cohort study (*n* = 14,036 adults; 1,052 cases) found that non-Hispanic Black-identifying participants positive for COVID-19 were more likely to be admitted to hospital than non-Hispanic White-identifying participants after adjusting for age, sex, comorbidities and income (*OR* = 2.67, *95% CI*: 1.30–5.47, *p*<0.01) [37].

Five studies involving bivariate analyses found no statistically significant differences in COVID-19 prevalence, clinical presentation, or outcomes across racial or ethnic groups [38–42]. A cross-sectional study of 305 people with COVID-19 admitted to hospitals in Georgia, USA, found that 83% identified as non-Hispanic Black (*n* = 247); however, compared to patients grouped as “other” race or ethnicity (including White, Asian, Hispanic and Pacific Islander; 17%, *n* = 50) there were no statistically significant differences in the proportions who received mechanical ventilation or died [42]. In a NYC, USA retrospective cohort study of patients with COVID-19 (*n* = 338), Toussie et al. did not find statistically significant differences in primary health outcomes of COVID-19 patients according to race or ethnicity [41]. The authors suggested, however, that Hispanic ethnicity was an independent predictor of having more severe chest x-ray findings among admitted patients (*n* = 145, *OR* = *not reported*, 95% CI: *not reported*, *p* = 0.03*)* [41].

Fifteen studies reported relative frequencies of participant race or ethnicity without testing for statistically significant differences in study outcomes [11, 43–56]; among these, most did not report comparisons with general population race or ethnicity demographics. Goyal et al studied the clinical characteristics of 393 COVID-19 patients from New York City, USA and found the majority of cases were non-White [52]. H. Sun et al described the race and ethnicity of 30 palliative COVID-19 patients in NYC, USA and found most were of Hispanic origin (66.7%) [50]. A case series from NYC found that, of 18 COVID-19 cases with cardiac events, 50% were Hispanic [55]. Notably, Laurencin and McClinton found that 17.2% of people infected with (*n* = 3141) and 14.4% of those dying from (*n* = 96) COVID-19 in Connecticut were Black. The authors remarked that these frequencies are higher than the proportion of the Connecticut population that identifies as Black (12%, *n* = NR), though tests of heterogeneity were not conducted [48].

### Occupation

Sixteen studies identified the occupations of participants. Ten studies were conducted in China [57–66], five in the USA [67–71] and one in Spain [72]. In China, labourers, retail staff, agricultural workers and healthcare workers were more commonly represented among those infected. Fan et al suggested that the first wave of infection in Gansu Province may have stemmed from migrant labor workers returning from Wuhan, as 29.1% of COVID-positive patients were migrant workers (7/24; *p* = 0.009) [58]. Another study examined 26 admitted COVID-19 positive cases in Liaochang and found that 16 (61.5%) were retail staff, 11 of whom worked at the same supermarket [65]. Agricultural workers or farmers were represented in six Chinese studies of COVID-19 patients, with relative frequencies ranging from 7.7% to 54.6% [61, 62, 64–66, 73]. In bivariate analyses, Shi et al found differences in the proportion of people with mild and severe COVID-19 symptoms comparing agricultural, non-agricultural, retired and student occupational groups, with agricultural workers having the most severe cases (*p*<0.001) [73]. Among four studies examining COVID-19 clinical features and describing occupation of participants, two did not assess differences by occupation [61, 62] and two found no statistically significant differences in the severity of symptoms by occupation [64, 66]. In Wuhan, China, Chu et al reported that among 54 hospitalized medical staff with COVID-19, severe disease tended to be more common among those working in non-emergency clinical or non-clinical settings [57]. Wang X. et al found that 16 of 80 hospitalized frontline medical workers in Wuhan were “other” non-medical healthcare workers compared to doctors and nurses [63].

Blanco et al conducted a case series of five HIV positive patients in Spain and found that two patients were sex workers, one of whom was admitted to ICU [72]. In the USA, five CDC Morbidity and Mortality Weekly Reports present the prevalence of COVID-19 among employees of homeless shelters, correctional or detention facilities and meat processing facilities. For homeless shelter staff, 21.0% (8/38) of staff at three homeless shelters in King County, Washington were positive [70], and 11.0% (33/313) of staff at 19 homeless shelters in Boston, Seattle, San Francisco and Atlanta were positive [69]. For correctional or detention facility staff, a study by the CDC using data from 37 states reported 2,778 cases of COVID-19 among staff members, of whom 3% became hospitalized and 1.0% died [67]. Another CDC study on 46 correctional and detention facilities in Louisiana found 253 staff members were infected with COVID-19, 7.5% of whom were hospitalized and 1.6% died [68]. Dyal et al assessed the incidence of COVID-19 among workers in meat and poultry processing facilities in 19 states [71]. Of the 130,578 workers in 115 affected meat and poultry processing facilities, 3.0% tested positive (4,913 cases) and 0.4% died; the authors hypothesized that language barriers, overcrowded housing, overcrowded transportation and incentives to continue to work while ill limited effective infection control.

### Income and socioeconomic status

Five studies reported on income or proxies for income. Two studies from the UK examined the association between socioeconomic status and positive COVID-19 case incidence. Hastie et al used the Townsend score to assess socioeconomic deprivation, which incorporates measures of unemployment, non-car ownership, non-home ownership and household overcrowding [35]. The authors found that higher socioeconomic deprivation predicted COVID-19 positive status in a multivariable logistic regression model (highest vs. lowest Townsend quintile *OR* = 1.89; *95% CI* = 1.37–2.60; *p* <0.001). De Lusignan et al assessed socioeconomic deprivation using the English Index of Multiple Deprivation, which incorporates measures including income, employment, education, health, crime, barriers to housing and services and living environment [36]. In both univariate and multivariate analyses, people living in more deprived areas were more likely to test positive for COVID-19 (*OR* = 2.03, *95% CI*: 1.51–2.71, *p<*0.0001).

Three studies reported associations between income factors and COVID-19 outcomes [35–37]. Azar et al found that COVID-19 patients in a California healthcare system with Medicaid or who were uninsured were more likely to be admitted to hospital compared to those with commercial insurance (Medicaid: *OR* = 2.13; *95% CI*: 1.24–3.68, *p*<0.01; Self-Pay/Unknown: *OR* = 2.19; *95% CI*: 1.03–4.36, *p*<0.05) [37]. The same study found COVID-19 patients residing in higher income neighbourhoods were less likely than those residing in lower income neighbourhoods to be admitted to hospital (High income, fourth quartile: *OR* = 0.55, *95% CI*: 0.33–0.91, *p*<0.05; High income, Third quartile: *OR* = 0.24, *95% CI*: 0.12–0.46, *p*<0.001). Two studies only described the income or insurance status of participants with COVID-19 without testing for associations or drawing comparisons to general population income demographics [42, 74].

### Social isolation

Two studies assessed factors related to social isolation. In a UK study of 3,802 adults tested for COVID-19, the odds of a positive test were lower in households with two to eight people, compared to single-person households in a univariate analysis (*p*<0.0001), but this was no longer statistically significant after adjusting for sociodemographic, lifestyle and health related factors [36]. One study examined social capital in relation to sleep quality and mental health outcomes among 170 adults in central China isolating at home following confirmed or suspected COVID-19, or a known exposure [74]. Social capital was measured using the Personal Social Capital Scale 16, scored according to number and professions of friends, relatives, coworkers; social trust; and civil society, recreational and political participation. After adjusting for potential confounders, higher social capital scores were significantly associated with lower anxiety and stress (structural equation model coefficients: anxiety, β = 0.619, *p*<0.001; stress, β = 0.327, *p*<0.001) [74].

### Education

Two descriptive studies conducted in China examined education level of participants. Zhang et al surveyed 205 individuals to study mental health outcomes of populations affected by COVID-19 in Zhongshan [75]. Of the 57 individuals who reported having COVID-19, 30.9% had a junior-middle school education or less, 27.3% had a senior-middle school education and 41.8% had a college education or more. No statistically significant differences were observed by education level between patients who reported having COVID-19, were put under quarantine, or were non-infected members of the general public. The second study described the education level of 170 participants without examining differences in study outcomes [74].

### Food security

One study by Li et al examined the association between malnutrition and COVID-19 prevalence in elderly hospitalized patients with COVID-19 in Wuhan, China [76]. Of 182 study participants, 52.7% were malnourished, 27.5% were at risk of malnutrition and 19.8% were non-malnourished (*p* = 0.018). In their discussion, the authors reported that the level of malnourishment was higher in elderly COVID-19 patients than in elderly people with other health issues described by published literature.

### Housing status

Six studies assessed housing-related factors among COVID-19 patient populations. Three descriptive studies and one cohort study from the USA examined COVID-19 incidence and outcomes among people experiencing homelessness. Tobolowsky et al studied a COVID-19 outbreak among three homeless services sites in King County, Washington and found a positive COVID-19 diagnosis in 35 of 195 residents (18%) tested [70]. Mosites et al assessed COVID-19 in 19 homeless shelters in Boston, Seattle, San Francisco and Atlanta [69]. Of the 1,292 shelter residents, 292 tested positive (25%). One shelter in San Francisco had 66% of 95 residents test positive. Baggett et al studied COVID-19 prevalence among homeless shelter residents in Boston and found that, of the 408 residents tested, 147 (36%) had a positive test result [77]. Among people testing positive for COVID-19 in California, Azar et al found no statistically significant association between homelessness and hospital admissions [37].

Two studies examined the incidence and outcomes of COVID-19 in correctional and detention facilities. We classified these as related to housing status because the authors describe the challenges of infection control within correctional facilities in relation to housing: crowded dormitories, shared bathrooms, limited medical resources, limited quarantine space and daily entry and exit of staff and visitors [67, 68]. Wallace, Hagan et al examined national incidence of COVID-19 in 37 US jurisdictions that reported outcomes on correctional and detention facilities. Across 32 jurisdictions, 420 facilities had at least one case of COVID-19 [67]. They found 4,893 COVID-19 cases among incarcerated or detained persons, of whom 491 (10%) were hospitalized and 88 (2%) died. In a separate study of 144 correctional and detention Louisiana, Wallace, Marlow et al identified 489 laboratory-confirmed COVID-19 cases among incarcerated or detained persons, of which 47 (7.6%) were hospitalized and 10 (2%) died [68].

### Quality assessment

The overall quality of included studies was low. Among the studies that involved comparison between people with or without COVID-19, or compared health outcomes among people with COVID-19, risk of selection and confounding biases were most common (Table 2). This was most often due to the descriptive nature of analyses and small samples recruited over short periods of time, with limited information provided by authors to assist readers in evaluating the representativeness of samples. Eleven studies were at high risk of confounding (e.g. bivariate analyses), while seven had unclear risk of confounding (e.g. multivariable analyses accounting for sociodemographic, lifestyle and health-related confounders, but not other factors thought to be implicated in racial/ethnic differences in COVID-19 risk, such as employment in high-risk professions) [20, 38, 40, 54, 60, 62–64, 66, 74, 75]. Many of the case series or cross-sectional studies also relied on small sample sizes and similarly had risk of selection bias (Table 3); eight of these either provided insufficient detail for measurement methods for sociodemographic variable or outcomes, or insufficient detail of handling of missing data, and were therefore at risk of measurement error [11, 44, 48, 52, 57, 67, 71].

**Table 2:** Mixed methods assessment tool quality assessment matrix for quantitative non-randomized studies.

Author	Are research questions clear?	Do the collected data allow the research questions to be addressed?	Are the participants representative of the target population?	Are measurements appropriate regarding both the outcome & exposure?	Are there complete outcome data?	Are the confounders accounted for in the design and analysis?	During the study period, does the exposure occur as intended?	Legend: + Yes —No ? Unclear
Azar et al. 2020 [37]	+	+	+	?	+	?	+	
Baggett et al. 2020 [54]	+	+	+	+	+	—	+	
Dai et al. 2020 [62]	+	+	?	+	+	—	+	
de Lusignan et al. 2020 [36]	+	+	?	+	+	?	+	
Fan et al. 2020 [58]	+	+	?	?	+	+	+	
Gold et al. 2020 [42]	+	?	?	+	+	?	+	
Hastie et al. 2020 [35]	+	?	—	+	+	+	?	
Jia et al. 2020 [60]	+	+	?	+	+	—	+	
Lechien et al 2020 [47]	+	+	—	+	?	+	?	
Li et al. 2020 [76]	+	+	?	+	+	+	+	
Mehta et al. 2020 [56]	+	+	+	+	+	?	+	
Mosites et al. 2020 [69]	+	+	?	+	+	—	+	
Nobel et al. 2020 [39]	+	+	+	+	+	?	+	
Ouyang et al. 2020 [64]	+	?	?	+	+	—	+	
Shi et al. 2020 [73]	+	+	+	—	+	?	+	
Y. Sun et al. 2020 [38]	+	+	?	?	+	—	+	
Toussie et al. 2020 [41]	+	+	?	+	+	?	?	
R. Wang et al. 2020 [61]	+	+	?	+	+	—	+	
X. Wang et al. 2020 [63]	+	+	+	+	+	—	+	
Xiao et al. 2020 [74]	+	—	?	+	+	—	—	
Yan et al. 2020 [40]	+	+	+	+	+	—	+	
Yu et al. 2020 [66]	+	+	?	?	+	—	+	
Zhang et al. 2020 [75]	+	+	?	+	+	—	+	

**Table 3:** Mixed methods assessment tool quality assessment matrix for quantitative descriptive studies.

Author	Are research questions clear?	Do the collected data allow the research questions to be addressed?	Is the sampling strategy relevant to address the research question?	Is the sample representative of the target population?	Are the measurements appropriate?	Is the risk of nonresponse bias low?	Is the statistical analysis appropriate to answer the research question?	Legend: + Yes —No ? Unclear
Bangalore et al. 2020 [55]	+	+	?	—	+	+	+	
Blanco et al. 2020 [72]	+	+	+	—	+	+	+	
Burrer et al. 2020 [51]	+	+	+	+	+	+	+	
Chu et al. 2020 [57]	+	+	+	+	?	+	+	
COVID National Incident Room Surveillance Team 2020a [44]	+	+	+	+	?	—	+	
COVID National Incident Room Surveillance Team 2020b [45]	+	+	+	+	+	+	+	
Dyal et al. 2020 [71]	+	+	?	?	?	?	+	
Garg et al. 2020 [11]	+	+	+	+	+	?	+	
Gold et al. 2020 [42]	+	+	+	+	+	+	+	
Goyal et al. 2020 [52]	+	+	+	+	+	—	?	
Hasan & Narasimhan 2020 [43]	+	+	?	?	—	+	?	
Laurencin & McClinton 2020 [48]	+	?	+	+	?	?	+	
Richardson et al. 2020 [53]	+	+	+	+	+	+	+	
H. Sun et al. 2020 [50]	+	+	+	—	+	+	+	
Tobolowsky et al. 2020 [70]	+	+	+	+	+	—	+	
Tolia et al. 2020 [49]	+	+	+	?	+	?	+	
Wallace, Hagan et al. 2020 [67]	+	+	?	?	?	—	+	
Wallace, Marlow et al. 2020 [68]	+	?	+	?	?	?	+	
L. Wang et al. 2020 [65]	+	+	?	—	+	+	+	

## Discussion

In this rapid review we identified 42 peer-reviewed studies that included sociodemographic factors in analyses of COVID-19 incidence, clinical presentation, and prognosis. Most studies involved descriptive analyses, however more recent studies involving larger samples and multivariable analyses found key social determinants of health to be associated with COVID-19 incidence and outcomes. The strongest evidence of associations stems from three observational studies from the USA and UK which found associations between race and ethnicity, health insurance status, neighbourhood-level socioeconomic deprivation, and likelihood of COVID-19 positive status and COVID-19 hospital admission [35–37]. Limited evidence was available on other factors including occupation, educational attainment, housing status or food security.

While it remains possible that these associations could at least in part be explained by residual confounding and selection bias, the emergent findings are consistent with patterns observed during the H1N1 pandemic [22–24]. Adverse social conditions at the individual and community level, reinforced by systemic issues such as racism [78, 79], may increase the likelihood of both COVID-19 infection and poor COVID-19 disease outcomes. Low-income earners are more likely to hold essential sales and service jobs and live in crowded housing conditions where ability to maintain physical distance from others is limited, increasing risk of virus exposure and transmission [10, 80–82]. Across studies and settings, labourers, retail staff, agricultural workers, healthcare workers and people working in congregate settings (shelters, correctional facilities, meat processing facilities) were reported to be over-represented among those infected. Homeless shelters face similar challenges in preventing the spread of COVID-19, including overcrowding, limited access to facilities for maintaining basic hygiene, and high rates of underlying comorbidities among clients [69, 70, 77].

This rapid review had several limitations. As with many rapid reviews, the short review timeframe, combined with the emergent nature of COVID-19 literature, limited the breadth of our analysis [83–86]. However, rapid reviews and full systematic reviews conducted on the same topic often produce similar conclusions [86]. Further, we screened *all* indexed English-language literature on COVID-19 published during the search period, ensuring we captured eligible studies. We did not address all social determinants of health, but focused on the ones that were likely most relevant to COVID-19 [87]. Our search extended only to April 27, 2020, with records identified through supplementary sources up until June 25, 2020. Small sample sizes, cohorts restricted to people testing positive for COVID-19, and the use of descriptive statistical methods limited the inferences that could be drawn from most of the early studies we reviewed. However, a number of studies published more recently have addressed these limitations.

Studies published since June 2020 tend to support our findings of disparities in COVID-19 infection, hospitalization, and mortality by race or ethnicity [88–96], socioeconomic status and deprivation [88–90, 92, 93, 97], and housing insecurity [95, 96, 98, 99]. At least two recent studies did not find associations between race and mortality outcomes among those able to access hospital care [100, 101], contrary to findings of most other research, including this review. More recent studies have also examined a wider range of sociodemographic factors in relation to COVID-19 infection such as primary spoken language [96], and additional studies have examined those factors less often assessed in early reports, such as educational attainment [90, 93, 97], occupation [97, 102, 103], and marital status [93]. Contrasting the early findings from one study included in our analysis, at least two studies indicate that cohabitation and larger households are associated with COVID-19 infection and mortality [103, 104]. Food insecurity appears to remain an understudied factor in relation to COVID-19 incidence and outcomes. At the time of publication, we identified only one systematic review examining COVID-19 outcomes by ethnicity [105].

Among early reports, few studies collected data on the social determinants of health. Those that did were at high risk of bias and frequently had missing data was common, with incomplete or missing data for race or ethnicity reported by nineteen studies, with missing data ranging from 2.6% to 61% [11, 36–45, 48–54, 56]. To enhance availability of high-quality evidence for policymakers, we recommend that further large-scale prospective studies are complemented by knowledge sources from community health, social service and advocacy organizations. Studies initiated at the outset of *future* pandemics should endeavor to collect and asses individual-level data on social risk factors using standard tools, ensuring data collection, interpretation and subsequent actions taken are led by the communities most impacted. The literature on COVID-19 continues to expand rapidly [106], and future systematic reviews with meta-analyses will be required to fully understand the magnitude of any effects and predictive utility of sociodemographic factors related to COVID-19 incidence and outcomes.

## Supporting information

S1 File Database search strategies. (DOCX)

S2 File Expert contacts. A list of individuals contacted to refer additional articles on the social determinants of COVID-19 incidence and outcomes who consented to be named.
(DOCX)

S3 File Data extraction form. (DOCX)

S1 Table Review PICO framework. A breakdown of the criteria applied to articles to determine inclusion eligibility.
(DOCX)

S1 Checklist (DOC)
