# Supplementary material for: Social determinants of COVID-19 incidence and outcomes: A rapid review
Source: PLoS One. 2021 Mar 31;16(3):e0248336. doi: 10.1371/journal.pone.0248336 (PMC8011781; doi:10.1371/journal.pone.0248336)
Supplement: S1 File — (DOCX) [file pone.0248336.s001.docx]

**S1 File. Database Search Strategies**

**Search dates:** April 1, 2020; April 16, 2020; April 27, 2020

**Database:** Ovid MEDLINE: Epub Ahead of Print, In-Process & Other Non-Indexed Citations, Ovid MEDLINE® Daily and Ovid MEDLINE® <1946-Present>

**Search Strategy:**

--------------------------------------------------------------------------------

1 (nCov or 2019 ncov or novel coronavirus or novel corona virus or covid-19 or SARS-COV-2 or Severe Acute Respiratory Syndrome Coronavirus 2 or coronavirus disease 2019 or coronavirus pandemic or coronavirus epidemic or coronavirus outbreak or corona virus pandemic or corona virus epidemic or corona virus outbreak or corona virus disease 2019 or new coronavirus or new corona virus or new coronaviruses or novel coronaviruses or 2019 ncov or nCov 2019 or SARS Coronavirus 2).tw,kf.

2 (wuhan and (coronavirus or corona virus)).tw,kf.

3 (Severe Acute Respiratory Syndrome Coronavirus 2 or COVID-19).os,ps,rs,ox,px,rx,nm.

4 1 or 2 or 3

5 limit 4 to (english language and yr="2019 -Current")

**Ovid Database:** Embase Classic+Embase <1947 to 2020 April 23>

**Search Strategy:**

--------------------------------------------------------------------------------

1 (nCov or 2019 ncov or novel coronavirus or novel corona virus or covid-19 or SARS-COV-2 or Severe Acute Respiratory Syndrome Coronavirus 2 or coronavirus disease 2019 or coronavirus pandemic or coronavirus epidemic or coronavirus outbreak or corona virus pandemic or corona virus epidemic or corona virus outbreak or corona virus disease 2019 or new coronavirus or new corona virus or new coronaviruses or novel coronaviruses or 2019 ncov or nCov 2019 or SARS Coronavirus 2).tw.

2 (wuhan and (coronavirus or corona virus)).tw.

3 1 or 2

4 limit 3 to english language

5 limit 4 to yr="2019 -Current"

6 limit 5 to embase

**Ovid Database:** APA PsycInfo <1806 to April Week 3 2020>

**Search Strategy:**

--------------------------------------------------------------------------------

1 (nCov or 2019 ncov or novel coronavirus or novel corona virus or covid-19 or SARS-COV-2 or Severe Acute Respiratory Syndrome Coronavirus 2 or coronavirus disease 2019 or coronavirus pandemic or coronavirus epidemic or coronavirus outbreak or corona virus pandemic or corona virus epidemic or corona virus outbreak or corona virus disease 2019 or new coronavirus or new corona virus or new coronaviruses or novel coronaviruses or 2019 ncov or nCov 2019 or SARS Coronavirus 2).tw

2 (wuhan and (coronavirus or corona virus)).tw.

3 1 or 2

4 limit 3 to yr="2019 -Current"

**CINAHL Plus**

**Search History**

Interface - EBSCOhost Research Databases
Search Screen - Advanced Search
Database - CINAHL Plus with Full Text

| \| **#** \| **Query** \| **Limiters/Expanders** \| \| --- \| --- \| --- \| \| S5 \| S3 AND S4 \| Search modes - Boolean/Phrase \| \| S4 \| EM 20191201-20200427 \| Search modes - Boolean/Phrase \| \| S3 \| S1 OR S2 \| Limiters - English Language Search modes - Boolean/Phrase \| \| S2 \| (wuhan and (coronavirus or corona virus)) \| Search modes - Boolean/Phrase \| \| S1 \| (nCov or 2019 ncov or novel coronavirus or novel corona virus or covid-19 or SARS-COV-2 or Severe Acute Respiratory Syndrome Coronavirus 2 or coronavirus disease 2019 or coronavirus pandemic or coronavirus epidemic or coronavirus outbreak or corona virus pandemic or corona virus epidemic or corona virus outbreak or corona virus disease 2019 or new coronavirus or new corona virus or new coronaviruses or novel coronaviruses or 2019 ncov or nCov 2019 or SARS Coronavirus 2) \| Search modes - Boolean/Phrase \| |
| --- | --- | --- | --- | --- | --- | --- | --- | --- | --- | --- | --- | --- | --- | --- | --- | --- | --- | --- |

**Cochrane Central Register of Controlled Trials (Wiley)**

(nCov or 2019 ncov or novel coronavirus or novel corona virus or covid-19 or SARS-COV-2 or Severe Acute Respiratory Syndrome Coronavirus 2 or coronavirus disease 2019 or coronavirus pandemic or coronavirus epidemic or coronavirus outbreak or corona virus pandemic or corona virus epidemic or corona virus outbreak or corona virus disease 2019 or new coronavirus or new corona virus or new coronaviruses or novel coronaviruses or 2019 ncov or nCov 2019 or SARS Coronavirus 2) or (wuhan and (coronavirus or corona virus)) in Title Abstract Keyword
