# Supplementary material for: Social determinants of COVID-19 incidence and outcomes: A rapid review
Source: PLoS One. 2021 Mar 31;16(3):e0248336. doi: 10.1371/journal.pone.0248336 (PMC8011781; doi:10.1371/journal.pone.0248336)
Supplement: S2 File — A list of individuals contacted to refer additional articles on the social determinants of COVID-19 incidence and outcomes who consented to be named. (DOCX) [file pone.0248336.s002.docx]

**S2 File. Expert Contacts.** A list of individuals contacted to refer additional eligible articles who consented to be named.

| 1 | Jason Agostino* | National Aboriginal Community Controlled Health Organisation (NACCHO) | Australia |
| --- | --- | --- | --- |
| 2 | Jessica Allen | University College London | United Kingdom |
| 3 | Onil Bhattacharyya | University of Toronto | Canada |
| 4 | David Blane | University of Glasgow | United Kingdom |
| 5 | Vanessa Brcic | University of British Columbia | Canada |
| 6 | Simon Capewell | University of Liverpool | United Kingdom |
| 7 | Caroline Costongs | EuroHealthNet | Belgium |
| 8 | Caroline Fichtenberg | Social Intervention Research and Evaluation Network (SIREN) | United States |
| 9 | Sharon Friel | Australian National University | Australia |
| 10 | Rick Glazier | University of Toronto, Canadian Institutes of Health Research | Canada |
| 11 | Laura Gottlieb* | University of California, San Francisco | United States |
| 12 | Jeannie Haggerty | McGill University | Canada |
| 13 | Kate Hunt | University of Stirling | United Kingdom |
| 114 | Stephen Hwang | University of Toronto, MAP Centre for Urban Health Solutions | Canada |
| 15 | Shreya Kangovi | University of Pennsylvania | United States |
| 16 | Jay Kaufman | McGill University | Canada |
| 17 | Ginetta Salvalaggio | University of Alberta | Canada |
| 18 | Arjumand Siddiqi | University of Toronto | Canada |
| 19 | Elizabeth A Sturgiss | Monash University | Australia |
| 20 | Chantal Verdonschot* | EuroHealthNet | Belgium |
| 21 | Sabrina Wong | University of British Columbia | Canada |
| *Secondary contact who was forwarded the email request from a primary contact | | |  |

**Email template used to solicit reports from colleagues**

Dear [colleague name],

The [Upstream Lab](https://upstreamlab.org/covid19review/) is conducting a systematic review of the COVID-19 literature to identify peer-reviewed studies that describe the social determinants of COVID-19 incidence and outcomes. We have completed a search of the indexed literature to April 27, identifying 32 quantitative studies (attached)

**We are seeking your help as an expert to identify additional peer-reviewed, empirical articles we may not have captured.** We are particularly interested in the following social determinants:

- Education, literacy
- Income, socioeconomic position, deprivation
- Employment, unemployment, and working conditions
- Housing conditions, homelessness, or incarceration
- Food security
- Race and racism
- Social isolation or social connectedness

**Please 'reply all' with papers you have come across,** and feel free to forward this message to your contacts.

We will certainly share our findings with you when this review is complete. Thank you so much for your time and support.

Andrew Pinto, MD CCFP FRCPC MSc

twitter: @AndrewDPinto

Director, [Upstream Lab](https://upstreamlab.org), MAP Centre for Urban Health Solutions, Li Ka Shing Knowledge Institute, Unity Health Toronto

Clinician-Scientist, St. Michael's Hospital

Associate Professor, Faculty of Medicine & IHPME & Dalla Lana School of Public Health, University of Toronto

Associate Director for Clinical Research, University of Toronto Practice-Based Research Network ([UTOPIAN](http://dfcm.utoronto.ca/landing-page/utopian))
