# Supplementary material for: Social determinants of COVID-19 incidence and outcomes: A rapid review
Source: PLoS One. 2021 Mar 31;16(3):e0248336. doi: 10.1371/journal.pone.0248336 (PMC8011781; doi:10.1371/journal.pone.0248336)
Supplement: S3 File — (DOCX) [file pone.0248336.s003.docx]

**S3 File. Data Extraction Form.**

| Study design | |
| --- | --- |
| Country | |
| Study resource context | - Urban - Rural - Community |
| Study setting | - Academic hospital - Community hospital - Outpatient primary care - Public health agency - Other (Homeless shelter, correctional or detention facility) |
| Geographic location of principle site | |
| Geographic location of additional sites | |
| Study observation start and end dates | |
| Participant population | |
| Sampling methods | |
| Participant numbers | - Eligible - Enrolled (including cases and controls, if relevant) - Lost to follow-up - Excluded from analysis (with reasons) |
| Study data start and end dates | |
| Analyses conducted | - Descriptive without or with significance testing - Correlation analysis - Analysis of variance - Bivariate or multivariate regression |
| Reported social determinants of interest | - Data collection (*i.e.* primary or secondary) - Data source (ex. health record) - Measurement (ex. marginalization index, self-report) - Level of data collect (*i.e.* individual, neighbourhood, or regional) - Percent missing responses |
| Reported outcomes of interest | - Data collection (*i.e.* primary or secondary) - Data source (ex. health record) - Measurement |
| Relevant result descriptions | |
