# Supplementary material for: Social determinants of COVID-19 incidence and outcomes: A rapid review
Source: PLoS One. 2021 Mar 31;16(3):e0248336. doi: 10.1371/journal.pone.0248336 (PMC8011781; doi:10.1371/journal.pone.0248336)
Supplement: S1 Table — A breakdown of the criteria applied to articles to determine inclusion eligibility. (DOCX) [file pone.0248336.s004.docx]

**S1 Table. *Review PICO Framework.*** A breakdown of the criteria applied to identify articles for inclusion.

| **PICO** | **Description** |
| --- | --- |
| **P**opulation(s) | Adults (18 years or older) with a presumptive or confirmed diagnosis of COVID-19 |
| **E**xposure(s) | Social determinants of health; specifically, race, income, educational attainment, employment status and working conditions, and social isolation |
| **C**omparator | Not applicable |
| **O**utcome(s) | COVID-19 infection incidence, reported as confirmed or presumptive diagnosis  Acute severe adverse events; specifically, mortality, incidence of acute respiratory distress syndrome (ARDS), incidence of multiple organ dysfunction syndrome (MODS), incidence of opportunistic infections, and incidence of cardiovascular events.  Health care utilization; specifically, hospitalization, ICU admissions, length of stay, proportion of patients requiring supplemental oxygen therapy or mechanical ventilation, length of time on supplemental oxygen therapy or mechanical ventilation.  Mental health outcomes  Exclusions: Laboratory measures (ex. C-reactive protein, ferrin, leukocyte counts, others); imaging or radiologic findings (ex. CT scan, chest x-ray, others) |
